# The recent advancements of ferroptosis in the diagnosis, treatment and prognosis of ovarian cancer

**DOI:** 10.3389/fgene.2023.1275154

**Published:** 2023-11-09

**Authors:** Biqing Chen, Liping Zhao, Rulin Yang, Tianmin Xu

**Affiliations:** The Second Hospital of Jilin University, Changchun, China

**Keywords:** ferroptosis, ovarian cancer, ferroptosis-related genes, immunotherapy, drug tolerance

## Abstract

Ovarian cancer affects the female reproductive system and is the primary cause of cancer related mortality globally. The imprecise and non-specific nature of ovarian cancer symptoms often results in patients being diagnosed at an advanced stage, with metastatic lesions extending beyond the ovary. This presents a significant clinical challenge and imposes a substantial economic burden on both patients and society. Despite advancements in surgery, chemotherapy, and immunotherapy, the prognosis for most patients with ovarian cancer remains unsatisfactory. Therefore, the development of novel treatment strategies is imperative. Ferroptosis, a distinct form of regulated cell death, characterized by iron-dependent lipid peroxidation, differs from autophagy, apoptosis, and necrosis, and may hold promise as a novel cell death. Numerous studies have demonstrated the involvement of ferroptosis in various conventional signaling pathways and biological processes. Recent investigations have revealed the significant contribution of ferroptosis in the initiation, progression, and metastasis of diverse malignant tumors, including ovarian cancer. Moreover, ferroptosis exhibits a synergistic effect with chemotherapy, radiotherapy, and immunotherapy in restraining the proliferation of ovarian cancer cells. The aforementioned implies that ferroptosis holds considerable importance in the management of ovarian cancer and has the potential to serve as a novel therapeutic target. The present review provides a comprehensive overview of the salient features of ferroptosis, encompassing its underlying mechanisms and functional role in ovarian cancer, along with the associated signaling pathways and genes. Furthermore, the review highlights the prospective utility of ferroptosis in the treatment of ovarian cancer.

## 1 Introduction

Ovarian cancer affects the female reproductive system and is the primary cause of cancer related mortality globally (Siegel et al., 2016). Annually, approximately 240,000 new cases of OC are reported (Bray et al., 2018), and 150,000 women succumb to this disease (Lheureux et al., 2019a). OC ranks fifth among the leading causes of cancer-related death in women globally, accounting for 4.4% of such deaths (Torre La et al., 2018). Ovarian cancer is a complex disease characterized by high heterogeneity and the presence of diverse subtypes. Epithelial ovarian cancer represents the majority of cases, accounting for 50%–70% of all cases, and can be further classified into type I, type II, and borderline type (Koshiyama et al., 2014). Type I tumors, which comprise 28% of ovarian cancer cases, encompass a range of histological subtypes, including mucinous carcinoma (MC), low-grade serous carcinoma (LGS), endometrioid carcinoma (END), and clear cell carcinoma (CCC) (Kopper et al., 2019). Type II tumors, which encompass high-grade serous ovarian cancer (HGSOC), constitute 57% of ovarian cancer cases and are responsible for 70%–80% of deaths among patients with this disease (Cancer Genome Atlas Research, 2011). The absence of distinctive clinical symptoms for ovarian cancer results in approximately 70% of patients being diagnosed at an advanced stage, with metastatic lesions extending beyond the ovary (Torre L. A. et al., 2018). This presents a significant challenge to clinical management and imposes a substantial economic burden on both patients and society. The prevailing conventional therapy for ovarian cancer entails a blend of cytoreductive surgery and platinum- and taxane-based combination chemotherapy (Aletti et al., 2007). Despite the initial efficacy of these interventions, a significant proportion of patients (70%–80%) experience relapse (Matulonis et al., 2016) and eventually develop resistance to chemotherapy (Patch et al., 2015). Empirical data indicates that approximately 25% of women encounter recurrence of ovarian cancer resistance within 6 months of the primary treatment (Reid et al., 2017). In recent years, advancements in surgical techniques, chemotherapy, and immunotherapy have led to an improvement in the overall survival rate of individuals diagnosed with ovarian cancer. However, despite these advancements, the majority of patients with ovarian cancer continue to experience unsatisfactory survival outcomes, with a 5-year overall survival rate of approximately 30% (Yeung et al., 2015). The absence of reliable diagnostic biomarkers and therapeutic targets for ovarian cancer underscores the pressing need to identify new and effective treatment options for this patient population. The identification of crucial predictive biomarkers and precise mechanisms of action is imperative in devising efficacious treatment strategies for ovarian cancer patients, thereby significantly enhancing their survival rates.

Recent research has demonstrated that the induction of ferroptosis can impede the proliferation of various tumor cells, including ovarian cancer (Sun et al., 2021). This process is regarded as a natural mechanism for suppressing tumors and plays a crucial role in the treatment of numerous types of cancer (Guo et al., 2018; Angeli et al., 2019; Wang et al., 2019a; Lang et al., 2019). The presence and advancement of diverse tumors are intricately linked to the concentration of intracellular Fe2+, encompassing lung cancer, liver cancer, and ovarian cancer (Stockwell et al., 2017a). Multiple investigations have indicated that the induction of ferroptosis is intimately associated with the progression of tumors. Furthermore, various studies have demonstrated the collaborative impact of ferroptosis in conjunction with chemotherapy, radiotherapy, and immunotherapy in impeding the proliferation of ovarian cancer cells. Basuli et al. have reported a marked increase in anomalous iron accumulation in high-grade serous ovarian cancer tissues compared to normal ovarian tissues (Basuli et al., 2017a). This finding, in light of the association between iron accumulation and ferroptosis, implies a potential correlation between ferroptosis and the advancement of ovarian cancer. A multicenter randomized controlled trial utilizing sorafenib and topotecan as maintenance therapy has demonstrated a noteworthy enhancement in progression-free survival among platinum-resistant ovarian cancer patients through the induction of ferroptosis (Sun et al., 2017; Chekerov et al., 2018). Moreover, contemporary empirical data has demonstrated the implication of ferroptosis in the modulation of tumor inception, progression, and dissemination. It is widely acknowledged that the conventional therapy for ovarian cancer entails the administration of platinum-based chemotherapy in conjunction with paclitaxel, which exerts a significant reduction in the quantity of malignant cells and enhances the prognostic outcome of patients. Nevertheless, a subset of patients may develop resistance to the treatment or experience a diverse range of unfavorable reactions subsequent to chemotherapy. Numerous studies have demonstrated the susceptibility of cancer cells to ferroptosis induction (Hangauer et al., 2017). Interestingly, recent investigations have revealed that the induction of ferroptosis in cancer cells can augment the anti-tumor effects of chemotherapy (Cheng et al., 2021). Additionally, radiotherapy has been established as an efficacious treatment modality for recurrent or refractory ovarian cancer, and research has shown that the synergistic enhancement of anti-tumor effects can be achieved by inducing ferroptosis in ovarian cancer cells in conjunction with radiotherapy (Lang et al., 2019). In contemporary times, the efficacy of immunotherapy in the treatment of cancer has gained widespread recognition. However, the limited application of immunotherapy drugs and the fact that only a third of ovarian cancer patients respond to them have prompted the exploration of alternative approaches (Liang et al., 2019a). In this regard, targeting ferroptosis in ovarian cancer cells presents a viable option for the treatment of ovarian cancer. This article provides an overview of the fundamental principles of ferroptosis, its role in ovarian cancer, and the potential therapeutic applications of ferroptosis in the management of ovarian cancer.

## 2 Mechanisms associated with ferroptosis

### 2.1 Characteristics of ferroptosis

**FIGURE 1:**
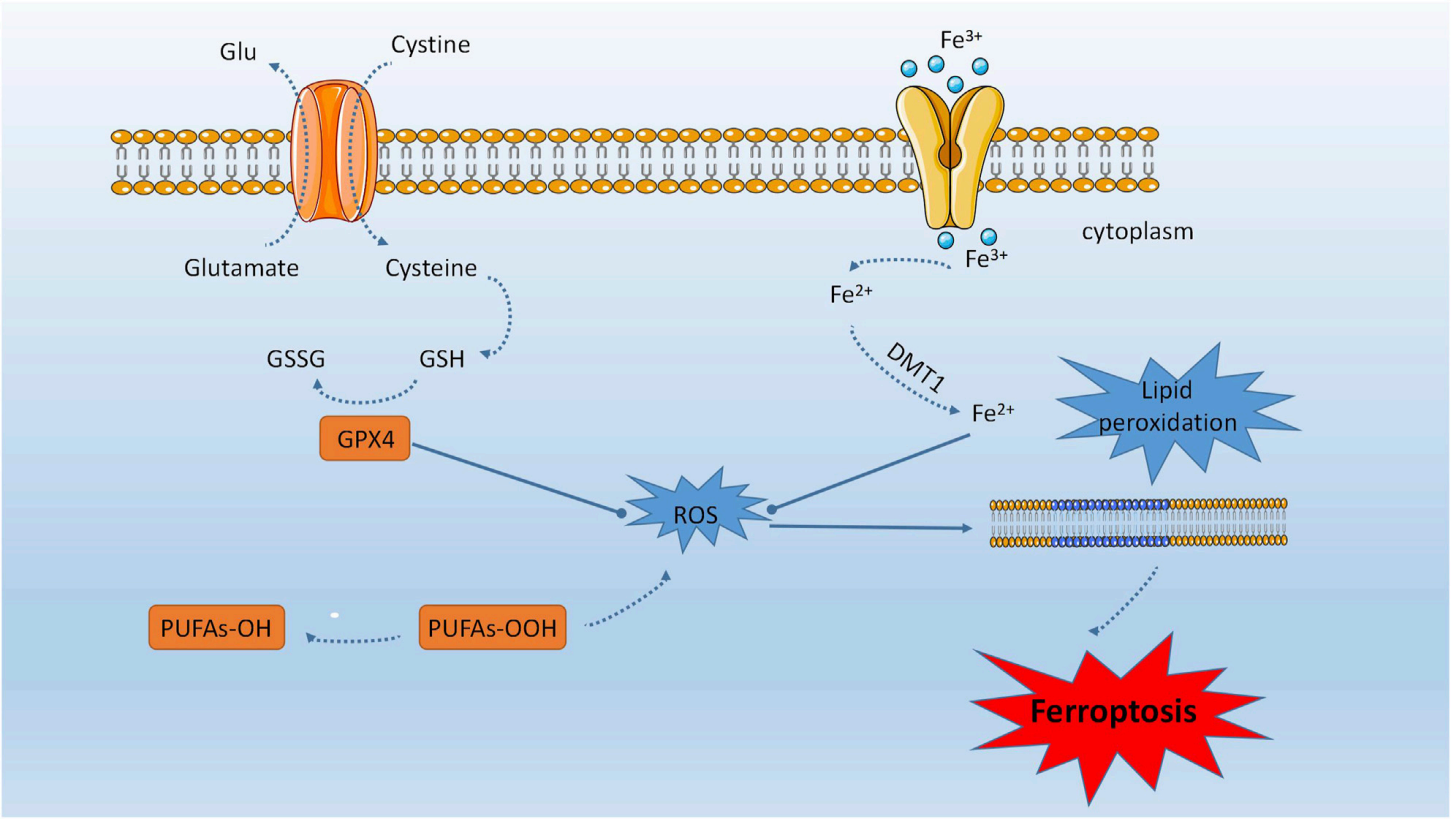
Ferroptosis is distinguished by the accumulation of unbound iron within cells, which can result in an overabundance of Fe2+ ions that participate in the Fenton reaction and generate hydroxyl radicals. The manifestation of ferroptosis is intricately linked to cysteine, lipid, and iron metabolism. The process of ferroptosis encompasses the Fenton reaction, system Xc-, and GPX4. The accumulation of peroxides of phospholipids containing polyunsaturated fatty acids (PUFA-PLs) is responsible for the onset of ferroptosis, as it disrupts cellular defense mechanisms.

Iron is an essential trace element that plays a crucial role in maintaining metabolism, such as participating in oxygen transport, DNA biosynthesis, and ATP synthesis. Iron is also an important mediator in regulating cell death. In 2012, Dixon et al. proposed iron-dependent regulated cell death, known as iron dependent death (Dixon SJ. et al., 2012). Recently, researchers have discovered that the characteristic of iron dependent death is the accumulation of lipid peroxidation induced by the iron ion pool (Dong et al., 2024). This finding could be utilized for selectively eliminating damaged cells and malignant tumor cells. Ferroptosis is distinguished by the accumulation of free iron within cells, which can lead to excess Fe2+ ions participating in the Fenton reaction and generating hydroxyl radicals. Insufficient antioxidant capacity in cells can result in an increase of hydroxyl radicals, leading to an oxidative stress response that causes membrane lipid peroxidation, elevated intracellular ROS, and mitochondrial dysfunction (Li et al., 2020). The onset of ferroptosis is intricately linked to cysteine metabolism (Yang et al., 2014), lipid metabolism (Yang and Stockwell, 2016), and iron metabolism (Gao et al., 2015), and involves the Fenton reaction, system Xc-, and GPX4 (Yagoda et al., 2007). The occurrence of ferroptosis is attributed to the buildup of phospholipid peroxides that contain polyunsaturated fatty acids (PUFA-PLs), which subsequently interfere with the cellular defense mechanisms (Hassannia et al., 2019a) (refer to Figure 1).

Iron plays a crucial role in the execution of iron dependent death. Transferrin receptor protein 1 (TFR1) on the cell membrane recognizes transferrin (TF) and forms a complex with Fe3+ from serum. Intracellular Fe3+ is first reduced by Six-transmembrane epithelial antigen of the prostate (STEAP3) in the cell nucleus, and then enters the cytoplasm via SLC11A2. When the expression of TFR1 increases, excessive accumulation occurs in the cytoplasm, leading to the generation of hydroxyl radicals and reactive oxygen species, triggering the Fenton reaction. This reaction damages the cell membrane, normal DNA, and proteins, ultimately resulting in iron dependent death. Ferritin, which stores excess iron in the cellular iron pool, exerts a universal anti-iron dependent death effect (Ma et al., 2022). Fe2+ participates in mitochondrial activities, such as oxygen transport, energy metabolism, and ferritin synthesis. When the expression of ferritin decreases, the cytoplasmic Fe2+ level increases, enhancing iron utilization. On one hand, Fe2+ generates a large amount of hydroxyl radicals and ROS through the Fenton reaction. On the other hand, it inhibits the synthesis of iron-sulfur clusters and promotes iron dependent death. Iron overload induces a significant increase in ferritin expression and leads to iron dependent death. Recent studies have shown that Amferon (AM5), a synthetic derivative that accumulates and sequesters iron in lysosomes, exhibits more effective and selective activity against breast cancer stem cells. However, the paradoxical situation of iron depletion triggers the degradation of lysosomal ferritin, further increasing the iron load in this organelle. Iron-mediated generation of reactive oxygen species promotes lysosomal membrane permeabilization and activates cell death pathways consistent with iron dependent death. These findings reveal iron and iron-mediated processes as potential targets for the treatment of these cells (Mai et al., 2017).

Abnormal lipid metabolism leads to lipid peroxidation, which is fatal to cells and contributes to cellular iron dependent death. Polyunsaturated fatty acids (PUFAs) are highly susceptible to lipid peroxidation and play a crucial role in cellular iron dependent death. Phosphatidylethanolamines (PEs) containing elongation products such as arachidonic acid (C20:4) or adrenic acid (C22:4) primarily promote lipid oxidation processes involved in cellular iron dependent death, making them potential targets for regulating iron dependent death. ACSL4 and LPCAT3 are enzymes associated with the composition and remodeling of polyunsaturated fatty acids in cellular membranes. Knocking out these enzymes may reduce the levels of lipid peroxidation substrates and enhance cellular resistance to iron dependent death. Reactive oxygen species (ROS) are molecules capable of damaging DNA/RNA, proteins, and lipids, ultimately leading to cell death. In the early stage of iron dependent death, ROS, particularly the accumulation of phospholipid hydroperoxides, play a critical role. Phosphatidylethanolamines (PEs) are major substrates for oxidation in the process of iron dependent death. Glutathione peroxidase 4 (GPX4) is a key enzyme in iron dependent death that can reduce the production of lipid hydroperoxides (PE-AA-OH and PE-AdA-OH). Therefore, GPX4 is a target for manipulating iron dependent death and can be regulated through inhibitors (such as RSL3), genetic methods (such as Cre recombinase), or by modulating substrate levels (such as glutathione depletion). GPX4 and glutathione play vital roles in protecting the cell membrane from lipid peroxidation caused by iron dependent death. Proteomic analysis has shown that overexpression of GPX4 in cancer cells can suppress iron dependent death mediated by the transcription factor RSL3, while GPX4 deficiency increases cellular sensitivity to iron dependent death. The synthesis and activity of GPX4 are influenced by the essential trace mineral selenium. Selenium is necessary for the proper functioning of GPX4, and supplementation can enhance GPX4 expression levels, thereby protecting cells from iron dependent death damage (Xu et al., 2023).

Peroxisomes, cellular organelles, promote cellular sensitivity to iron dependent death by synthesizing polyunsaturated ether phospholipids. The changes in lipid abundance are associated with alterations in sensitivity to iron dependent death during cellular state transitions. Using a whole-genome CRISPR-Cas9 screening of suppressors, it has been identified that oxidized peroxisomes are key contributors to the sensitivity of iron dependent death in human renal and ovarian cancer cells. Lipidomics analysis reveals that peroxisomes promote iron dependent death by synthesizing polyunsaturated ether phospholipids (PUFA-ePLs). PUFA-ePLs act as substrates for lipid peroxidation, inducing iron dependent death. Initially iron dependent death-sensitive cancer cells can transition to a state of resistance to iron dependent death in mice, which is correlated with a significant downregulation of PUFA-ePL abundance. Further investigations have shown that the pro-iron dependent death effects of PUFA-ePLs are not limited to tumor cells but can extend to other cell types, including neurons and cardiomyocytes. This study uncovers the significant role of peroxisomes-ether phospholipid axis in modulating susceptibility and evasion of iron dependent death, emphasizing that PUFA-ePLs represent a unique functional lipid category that is dynamically regulated during cellular state transitions, suggesting multiple regulatory nodes as potential therapeutic targets for diseases involving iron dependent death (Zou et al., 2020a).

Glutathione, one of the substrates for GPX4, may be influenced by the availability of cysteine during its biosynthesis. Therefore, amino acid metabolism is closely linked to the regulation of cellular iron dependent death. Cysteine transport relies on the cysteine/glutamate antiporter system Xc-, which consists of the regulatory subunit solute carrier family 3 member 2 (SLC3A2) and the catalytic subunit solute carrier family 7 member 11 (SLC7A11). The input of cysteine can be inhibited by erastin, thereby inducing iron dependent death. OTU deubiquitinase with ubiquitin aldehyde binding 1 (OTUB1), a member of the OTU family, has been found to stabilize SLC7A11. Inactivation of OTUB1 sensitizes ovarian cancer cells to iron dependent death. Additionally, p53 and breast cancer type 1-associated protein 1 (BAP1) can downregulate the expression of SLC7A11, leading to iron dependent deathin cancer cells. Multidrug resistance protein 1 (MRP1) can reduce intracellular levels of glutathione (GSH). Furthermore, cancer cells with high expression of MRP1 are sensitive to iron dependent deathinducers targeting GSH metabolism. It is widely accepted that iron dependent deathis a feasible process and can be effectively controlled through metabolic modulation (Xu et al., 2023).

Cellular ferroptosis is distinct from autophagy, apoptosis, and necrosis in terms of morphology and biochemistry (Xu et al., 2023). Morphologically, cells undergoing ferroptosis exhibit reduced mitochondrial volume, heightened mitochondrial bilayer membrane density, diminished or absent mitochondrial cristas, and disrupted mitochondrial outer membrane (Xu et al., 2023). In contrast to cellular necrosis, ferroptosis does not present with cytoplasmic and organelle swelling or plasma membrane disruption. In contrast to apoptosis, ferroptosis does not manifest chromatin condensation or the generation of apoptotic bodies. Similarly, unlike autophagy, ferroptosis does not demonstrate the formation of conventional autophagosomes (Mai et al., 2017). Furthermore, the identification of ferrodead cells can be accomplished through microscopic examination due to their distinctive balloon-shaped phenotype, which is characterized by the emergence of a transparent spheroid cell consisting predominantly of vacuous cytoplasm (Agmon et al., 2018).

Several scholars have discovered that ferroautophagy has the ability to regulate intracellular iron levels, engage in the excessive accumulation of ROS caused by the Fenton reaction, and subsequently participate in various pathological processes such as cell proliferation, differentiation, and programmed cell death through the mediation of cell ferroptosis. Additionally, ferritophagy is a specific process that involves the recognition and transportation of ferritin to lysosomes for autophagic degradation, leading to the release of free Fe2+ in both normal and tumor cells. Previous research has demonstrated that ferroautophagy plays a role in regulating intracellular Fe2+ levels and is involved in cellular processes such as proliferation, differentiation, and apoptosis. Dysregulation of ferritophagy can result in disruptions to intracellular iron homeostasis. For instance, insufficient ferritophagy during erythropoiesis can cause a reduction in intracellular iron content and lead to anemia, whereas excessive ferritophagy can result in an overabundance of intracellular Fe2+, leading to the accumulation of reactive oxygen species and ultimately, cell ferroptosis. Basuli et al. formulated a genetic model linked to ovarian cancer initiating cells, wherein they observed that the development of ovarian cancer was linked to a decrease in Fe2+ efflux pump activity and an increase in Fe2+ transporter expression (Basuli et al., 2017a). Consequently, certain scholars have proposed that directing therapeutic interventions towards Fe2+ metabolism during the initial stages of ovarian cancer cells could serve as a promising strategy to eliminate cancer cells.

Tumor stem cells are a type of cells in tumors that possess the ability to initiate tumor formation and self-renewal, and they are the main cause of resistance to traditional anticancer drugs. Recent studies have found that iron dependent deathinducers can selectively induce cell death in cancer stem cells within tumors, thus favoring the elimination of cancer stem cells and overcoming tumor resistance. Cancer cells often reduce iron release by upregulating transferrin receptor 1 (TfR1) or inhibiting membrane iron transporter, or increase iron uptake through these mechanisms to sustain cell proliferation. Studies on tumor stem cells have shown an increase in iron content in certain types of tumors, indicating the involvement of altered iron transport in tumor stem cell proliferation. Researchers have observed higher levels of TfR1 expression in breast cancer stem cells and ovarian cancer stem cells. In human tumors, changes in ferritin expression in tumor stem cells have been associated with poor prognosis. Moreover, iron is also involved in the accumulation of reactive oxygen species (ROS) through increased iron uptake and inhibition of iron chelators, which mediates the Fenton reaction. The connection between abnormal iron metabolism in tumor stem cells and iron dependent deathhighlights the potential of iron dependent deathas a promising target in combating tumor stem cells, enhancing treatment efficacy, and reversing drug resistance (Xu et al., 2023).

Various studies have indicated that pathways involved in iron dependent deathplay significant roles in the maintenance and survival of Cancer Stem Cells (CSCs). Maximizing treatment benefits and enhancing cancer therapies can be achieved through therapeutic combinations targeting cellular iron dependent death. However, there are factors that need to be considered. Firstly, different cancer cell lines may exhibit varying sensitivity to iron dependent death. Some gene expression markers may not be applicable to all tumor types, thus careful selection is important among different tumor types. Secondly, controlling exogenous factors such as iron, selenium, oxygen, cysteine, glutathione, and polyunsaturated fatty acids is crucial as they may influence the sensitivity of iron dependent deathin CSCs. Thirdly, since iron dependent deathis regulated by a complex network, targeting a single checkpoint may not be sufficient. Fourthly, interfering with multiple processes involved in iron dependent deathsimultaneously is necessary. Lastly, iron dependent deathis just one form of cell death. In a particular tumor, there may be several different forms of cell death, and in such cases, inducing iron dependent deathalone may not have a significant lethal effect on CSCs. Therefore, a combined intervention strategy may be more effective in enhancing the elimination of CSCs.

### 2.2 Ferroptosis-related genes and signaling pathways

Ferroptosis involves multiple signaling pathways, including the Hippo pathway (Zou et al., 2020a), MAPK pathway, and P53 pathway (Jiang et al., 2015a; Hattori et al., 2017). A growing number of ferroptosis-related genes (FRGs) have been identified, such as SLC7A11, GPX4, Nrf2, ATF4, FSP1, and others (Chen et al., 2017; Xie et al., 2017; Bersuker et al., 2019; Dodson et al., 2019). Recent research has demonstrated that the ferroptosis-related gene PRNP functions as a tumor suppressor in ovarian cancer, and its abnormal expression and function may serve as a potential biomarker for the diagnosis, prognosis, and immunotherapy response of this disease (Hu et al., 2022). To date, researchers have identified four primary pathways for cellular resistance to ferroptosis. Of these, three are predominantly localized in the plasma membrane (SLC7A11/GPX4, FSP1/CoQ10, and GCH1/BH4), while DHODH/CoQ10 primarily functions to eliminate mitochondrial lipid peroxidation (Kuhl et al., 2012). As research on ferroptosis continues to deepen, an increasing number of studies have demonstrated its significant association with various human diseases, including cancer, degenerative diseases, cancerization, stroke, cerebral hemorrhage, trauma, brain injury, ischemia-reperfusion injury, and renal degeneration (Stockwell et al., 2017b). Recent research has demonstrated that ferroptosis possesses immunogenic properties, and the induction of adaptive immune responses in cancer cells undergoing ferroptosis during the early stages can result in anti-tumor effects (Efi et al., 2020). Furthermore, immunotherapy has exhibited significant clinical efficacy in the management of ovarian cancer. Currently, there exists a paucity of research on the clinical relevance of ferroptosis-associated genes in the areas of ovarian cancer diagnosis, prognosis, immunotherapy, and chemotherapy. However, recent progress in the targeting of ferroptosis-associated genes (FRGs) or their signaling pathways to elicit ferroptosis in the context of cancer diagnosis and treatment has garnered significant interest (Liang et al., 2019b).

CD44 is a transmembrane glycoprotein that is involved in various biological processes dependent on epigenetic plasticity, including development, inflammation, immune response, wound healing, and cancer progression. It is commonly referred to as a cell surface marker, but recent studies have demonstrated its crucial role in mediating the endocytic uptake of iron-bound hyaluronic acid in tumor cell lines, primary cancer cells, and tumors. This polysaccharide-mediated iron uptake mechanism is enhanced during epithelial-mesenchymal transition, where iron acts as a metal catalyst controlling the inhibitory histone marks of mesenchymal gene expression through demethylation. CD44 itself is transcriptionally regulated by nuclear iron, which modulates its expression through a positive feedback loop, contrary to the negative regulation of transferrin receptor by excess iron. Epigenetic plasticity can be altered by perturbing cellular iron homeostasis. This study revealed a widespread iron uptake mechanism present in the cellular mesenchymal state, elucidating the central role of iron as a regulator of epigenetic plasticity (Müller et al., 2020). Additionally, CD44 also mediates the uptake of various metals, including copper. It has been found that a chemically reactive copper pool exists in the mitochondria of inflammatory macrophages, where copper (II) activates the peroxide-coupled NAD(H) redox cycle. The maintenance of the NAD(H) pool is crucial for metabolic and epigenetic programming of the inflammatory state. Inducing a reduction in the NAD(H) pool by targeting mitochondrial copper (II) with the dimeric biguanide compound LCC-12 leads to the modulation of macrophage activation, metabolism, and epigenetic state. This work emphasizes the central role of copper as a regulator of cellular plasticity and reveals a therapeutic strategy based on metabolic reprogramming and control of epigenetic cell states (Solier et al., 2023).

## 3 Progress of ferroptosis in the treatment of ovarian cancer

Globally, ovarian cancer is considered the most challenging gynecologic malignancy to manage (Müller et al., 2020). Despite the clinical validation of numerous novel anticancer agents, including molecular targeted drugs, bevacizumab or PARP inhibitors, and combination therapy, the overall survival rate of patients has not shown significant improvement due to the development of drug resistance in most ovarian cancer cells following initial treatment response (Biamonte et al., 2019; Rose et al., 2020). Reduced susceptibility to apoptosis is a crucial mechanism of acquired resistance, as evidenced by scholarly research (Solier et al., 2023). Consequently, novel therapeutic approaches have been investigated, yielding significant progress in the understanding of genetic determinants and biological processes of ferroptosis since its initial discovery. The correlation between ovarian cancer treatment and ferroptosis has garnered scholarly attention, with numerous studies reporting potential therapeutic strategies centered on cellular ferroptosis. The susceptibility of ferroptosis is modulated by diverse biological mechanisms, including p53 deficiency (Tarangelo et al., 2018), DNA damage signaling pathways (Chen et al., 2019a), metabolic alterations (Ding et al., 2018), and epithelial-mesenchymal transition (EMT) (), which are frequently disrupted in ovarian cancer. Consequently, the quest for pharmacological agents capable of inducing ferroptosis in ovarian cancer cells has emerged as a prominent research objective.

### 3.1 Therapies targeting ferroptosis-related signaling pathways and genes

Ferroptosis has emerged as a promising therapeutic avenue for various treatment-resistant cancers, as evidenced by recent literature (Battaglia et al., 2020). Preclinical investigations have demonstrated that ferroptosis inducers (FINs) can augment the chemosensitivity of ovarian cancer cells (Lin and Chi, 2020). Notably, a study has reported that KRAS mutation or amplification is present in approximately 13.9% of ovarian cancer patients (Ratner et al., 2010). KRAS has been implicated in ferroptosis and lipid biosynthesis (Mukhopadhyay et al., 2021), while other studies have suggested that mutated KRAS activates the NRF2 antioxidant pathway. The activation of glutaminolysis and glutamine deprivation by NRF2 results in the reduction of GPX4 levels, as indicated by recent research (Mukhopadhyay et al., 2020). These findings highlight the potential of targeting KRAS as a treatment strategy for ovarian cancer, given that KRAS mutation serves as a genetic determinant that sensitizes cancer cells to erastin-induced ferroptosis (Battaglia et al., 2020). Cells with KRAS mutations demonstrate elevated levels of transferrin receptor 1 (TFR1) and diminished expression of ferritin heavy chain (FtH), resulting in heightened cellular iron absorption, suppression of iron storage, and buildup of labile iron pool (LIP) (Mou et al., 2019). Subsequently, intracellular free Fe2+ initiates the Fenton reaction, leading to the generation of reactive oxygen species (ROS), rendering cells more vulnerable to ferroptosis (Lei et al., 2019). The upregulation of ABCB1 is recognized as a significant contributor to the ineffectiveness of cancer chemotherapy (Seborova et al., 2019). Erastin has been shown to enhance the responsiveness of ovarian cancer cells to docetaxel by reducing the drug efflux activity promoted by ABCB1 (Zhou et al., 2019a). The combination of erastin and docetaxel is posited as a potential strategy to expand the limited treatment options for chemoresistant ovarian cancer (Zhou et al., 2019a). Recently, Torti et al. have proposed that ovarian cancer cells exhibit heightened iron uptake and reduced iron efflux, rendering them more susceptible to erastin both *in vitro* and *in vivo* (Basuli et al., 2017b). Erastin has been found to decrease cystine import, resulting in REDOX imbalance through the reduction of intracellular glutathione levels (Dolma et al., 2003). Glutathione serves as a cofactor for glutathione peroxidase (GPX4), an enzyme responsible for breaking down the accumulation of lipid reactive oxygen species. Consequently, inhibition of GPX4, whether chemically or genetically induced, can trigger ferroptosis and lipid peroxidation (Yang et al., 2014). Previous research has demonstrated that the EMT activator ZEB1 regulates GPX4 levels, which may determine the susceptibility of resistant cancer cells to ferroptosis, indicating that GPX4 plays a significant role in ferroptosis (Viswanathan et al., 2017a; Hangauer et al., 2017). Alternatively, upregulation of NADPH oxidase can lead to the formation of lipid reactive oxygen species and ferroptosis (Dixon SJ. et al., 2012). To this end, imidazolone erastin (IKE) has been developed for *in vivo* use, owing to its high potency, solubility, and metabolic stability (Zhang Y. et al., 2019). Consequently, further optimization of this agent is warranted for future clinical application, with the aim of inducing ferroptosis to enhance the prognosis of patients with advanced ovarian cancer. Multiple studies have demonstrated that the NRF2/CBS activated anti-sulfur pathway exhibits resistance against erastin-induced ferroptosis (Liu N. et al., 2020). Further investigation is necessary to elucidate the role of NRF2 in inhibiting cell ferroptosis for effective clinical treatment of ovarian cancer patients. *In vitro* experiments have revealed that Artesunate (ART) induces a robust generation of reactive oxygen species (ROS), resulting in the inhibition of ovarian cancer cell proliferation and tumor growth in relevant animal models (Greenshields et al., 2017). The inhibition of SLC7A11 by the PARP inhibitor olaparib has been observed to synergistically promote ferroptosis in HEY and A2780 cells when administered with erastin or sulfasalazine (Hong et al., 2021a). Currently, FINs have only demonstrated efficacy in a specific subset of patients with ovarian cancer (Wang et al., 2020).

The SNAI2 gene, also referred to as Slug, encodes a zinc-finger protein that belongs to the SNAI family of transcription factors. Current research indicates that SNAI2 plays a significant role in cancer progression, including the activation of tumor-initiating cells and the facilitation of cell invasion and metastasis (Fan et al., 2020). Investigations have demonstrated that SNAI2 directly binds to the promoter and regulates SLC7A11 expression and the downregulation of SNAI2 may stimulate ferroptosis in ovarian cancer cells by directly targeting and suppressing SLC7A11, consequently impeding the advancement of ovarian cancer (Jin et al., 2022a).

Research has reported that superparamagnetic iron oxide nanoparticles (SPIONs) are well-known nucleic acid or drug carriers due to their controllability, excellent stability, and ease of modification. By inducing the accumulation of reactive oxygen species, SPIONs can lead to cellular oxidative stress. Treatment of cells with SPIONs significantly reduces the expression of tumor stem cell markers (CD117, NANOG, CD133, and SOX2), cell proliferation factors (KI67, CCND), autophagy-related factors (ATG3, ATG5, MAP1ALC3a, MAP1ALC3b, and MAP1ALC3c), and some negative regulators of ferroptosis, while significantly increasing the mRNA expression levels of cell death-related proteins (BAK1 and BID) and certain positive regulators of iron dependent death. These findings suggest that SPIONs inhibit the proliferation, invasion, and drug resistance of ovarian cancer stem cells, thereby reducing ovarian cancer recurrence and metastasis (Huang et al., 2020a).

MEX3A is a dual-function protein that contains both RING finger domains and RNA-binding domains. It has been identified as an important oncogenic factor, as it can inhibit p53-mediated cell death by iron, thereby promoting the development of ovarian cancer (Wang et al., 2023). These findings provide new insights into the mechanisms of ovarian cancer development and can contribute to the development of effective tumor treatment strategies that selectively target ovarian cancer expressing wild-type p53.

SKP2 serves as a pivotal regulator of cell cycle, senescence, and tumorigenesis (Cai et al., 2020), rendering it a promising therapeutic target for cancer. Furthermore, scholarly research has revealed that YAP governs the transcription of SKP2 mRNA, thereby facilitating the YAP-activated cell proliferation program and vulnerability to ferroptosis (Yang et al., 2021). Tumors activated by YAP or SKP2 may exhibit heightened sensitivity to ferroptosis and may respond favorably to ferroptosis-based therapeutic interventions (Cai et al., 2020). However, extant research has demonstrated that elevated levels of YAP or SKP2 can engender resistance to chemotherapy, thereby prompting conjecture regarding a plausible association between chemotherapy resistance and augmented ferroptosis sensitivity. This underscores the rationale for inducing ferroptosis as a means of eradicating treatment-resistant tumors.

Recent research has demonstrated that carboplatin therapy triggers the ACSL1/FSP1 anti-ferroptosis pathway, resulting in decreased sensitivity of cancer cells to platinum-based chemotherapy and enhanced cancer cell survival. However, the precise molecular mechanisms underlying platinum resistance in ovarian cancer necessitate further investigation. Consequently, this study identifies a potential therapeutic target for ovarian cancer and establishes a theoretical foundation for clinical interventions aimed at overcoming platinum resistance in ovarian cancer (Zhang Q. et al., 2023).

Studies have shown that SPIO-Serum can effectively induce lipid peroxidation, leading to the generation of a large amount of reactive oxygen species (ROS). It also promotes the downregulation of GPX4 and xCT, ultimately resulting in iron-dependent oxidative cell death. SPIO-Serum treatment disrupts cellular iron homeostasis by modulating iron uptake and causing mitochondrial cristae loss and outer mitochondrial membrane rupture. SPIO-Serum induces iron dependent deathin ovarian cancer cells by disrupting iron metabolism, mitochondrial function, and redox homeostasis, and it promotes cell iron dependent deathby increasing p53 expression (Zhang et al., 2021a). This research provides a theoretical basis for the development of iron-based nanoparticles as a novel therapeutic agent for ovarian cancer and offers new hope for clinical treatment of ovarian cancer patients.

Small extracellular vesicles (SEVs) are nanovesicles that originate from endosomes and facilitate intercellular communication at both physiological and pathological levels through the enrichment of nucleic acids, lipids, and proteins (Fafián-Labora and O’Loghlen, 2020). Prior research has indicated that extracellular vesicles play a crucial role in driving ferroptosis and increasing sEV secretion by removing intracellular iron (Yu M. et al., 2019; Brown et al., 2019). Furthermore, sEVs have the ability to modulate the metabolic state of cells by regulating glutathione metabolism and lipid peroxidation (Fafián-Labora et al., 2020). Hence, the utilization of sEV-mediated cell-to-cell communication presents a promising strategy in the fight against ovarian cancer, as evidenced by previous research (Alarcón-Veleiro et al., 2023).

Zhang et al. have demonstrated that the attenuation of the PKCβII-ACSL4 pathway effectively blocked cell ferroptosis by reducing the accumulation of lipid peroxidation (Zhang HL. et al., 2022). Additionally, Yi et al. have pointed out that the aberrant activation of PI3K-AKT-mTOR signaling protects cancer cells from oxidative stress and ferroptosis through SREBP1/SCD1-mediated lipogenesis (Yi et al., 2020a). These findings establish a theoretical foundation for targeting the ferroptosis pathway in the clinical treatment of ovarian cancer.

Elevated expression of TAZ mRNA has been linked to unfavorable prognosis in ovarian cancer, impacting migration, proliferation, treatment response, and EMT, as reported in sources (Jeong et al., 2013; Tan et al., 2015). ANGPTL4, a constituent of the angiopoietins family that modulates lipid and glucose metabolism (Santulli, 2014), is overexpressed in multiple human cancers, stimulated by diverse oncogenic pathways, and correlated with tumor metastasis (Tanaka et al., 2015; Baba et al., 2017). The activation of NADPH oxidase by ANGPTL4 has been observed to stimulate oncogenic ROS(Zhu et al., 2011). Additionally, it has been discovered that TAZ serves as a genetic determinant of ferroptosis through the regulation of ANGPTL4-NOX2, thereby highlighting its potential as a novel therapeutic target for ovarian cancer and other TAZ-activated tumors (Yang et al., 2020). The induction of ferroptosis may prove to be a promising therapeutic approach.

In recent times, an increasing number of experimental findings have demonstrated that microRNAs can function as both oncogenes and tumor suppressor genes, thereby exerting a significant influence on the proliferation, apoptosis, and differentiation of ovarian cancer cells (Berindan-Neagoe et al., 2014; Bagnoli et al., 2016; He et al., 2019). Recent investigations have also indicated that the suppression of miR-4715-3p, miR-9, and miR-137 can trigger ferroptosis in ovarian cancer cells (Luo et al., 2018; Zhang et al., 2018; Gomaa et al., 2019). The study revealed that miR-424-5p exerts a negative regulatory effect on ferroptosis in ovarian cancer cells through direct targeting of ACSL4. The overexpression of miR-424-5p results in the inhibition of ACSL4 by binding directly to its 3′-UTR, which subsequently reduces the occurrence of ferroptosis induced by erastin and RSL3. These findings present a novel therapeutic avenue for the management of ovarian cancer (Ma et al., 2021).

The study revealed that the combination of apatinib and olaparib effectively suppressed ferroptosis in ovarian cancer cells via the p53-mediated Nrf2 pathway (Kang et al., 2019). The tumor suppressor protein p53 has been shown to impede tumor progression by inducing ferroptosis, which it regulates through both transcriptional and post-translational mechanisms (Liang et al., 2019a; Kang et al., 2019). Additionally, p53 can facilitate the buildup of lipid reactive oxygen species and trigger ferroptosis by repressing the transcription of the glutamate/cystine antitransporter SLC7A11 (Jiang et al., 2015b). Furthermore, it has been demonstrated that p53 exerts a direct inhibitory effect on DPP4 in a transcription-independent fashion, thereby impeding the occurrence of ferroptosis (Xie et al., 2017; Tarangelo et al., 2018). This finding offers a theoretical foundation for the potential synergistic use of apatinib and olaparib in clinical settings (Hong et al., 2021b).

Carboxymethylated pachyman (CMP) is a carboxymethylated derivative of the polysaccharide extracted from Poria cocos (also known as Fu Ling). CMP has been found to possess immunomodulatory, anti-tumor, and antioxidant activities. However, whether CMP can be beneficial for the treatment of ovarian cancer is still not fully understood. Studies have revealed that CMP induces iron dependent death in ovarian cancer cells by inhibiting the Nrf2/HO-1/xCT/GPX4 pathway (Jin et al., 2022b). CMP may hold great potential in ovarian cancer treatment by inducing iron dependent death. Further research is needed to fully explore the therapeutic applications of CMP in ovarian cancer.

SLC7A11 serves as the catalytic subunit for the amino acid transport system Xc−, responsible for the uptake of extracellular cystine. This cystine is subsequently reduced to cysteine within the cytosol, serving as the rate-limiting precursor for glutathione (GSH) biosynthesis (Stockwell et al., 2017a). GSH, a potent scavenger of lipid peroxides, is a crucial cofactor for the selenoenzyme glutathione peroxidase 4 (GPX4), which effectively reverses phospholipid peroxidation and protects cells from ferroptosis (Cao et al., 2019). Hence, the SLC7A11-GSH system plays a crucial role in safeguarding cells against ferroptosis, and the activation of this system through ferroptosis inducing agents (FINs) to instigate ferroptosis or the induction of ferroptosis in conjunction with chemoradiotherapy presents a promising strategy for cancer treatment (Dixon S. J. et al., 2014; Stockwell et al., 2017a). In part, PARP inhibitors can facilitate ferroptosis by impeding SLC7A11-mediated GSH biosynthesis. Furthermore, the investigation has revealed that the utilization of olaparib in conjunction with FINs may serve as a potentially efficacious therapeutic approach for BRCA wild-type ovarian cancer (Hong et al., 2021c). Additionally, SLC7A11, a fundamental regulator of the cellular ferroptosis defense system, presents itself as a promising target for cancer therapy (Dixon S. J. et al., 2014; Hu et al., 2020).

Lidocaine, a local anesthetic drug that is a derivative of cocaine, is extensively employed in clinical settings for its antimicrobial, anti-inflammatory, and local skin anesthesia properties, among others (Cizmarikova et al., 2020). Recent research has demonstrated that lidocaine promotes cell ferroptosis by up-regulating miR-382-5p and suppressing SLC7A11 levels. Consequently, the potential therapeutic efficacy of lidocaine in the management of ovarian cancer warrants further elucidation (Sun et al., 2021).

Ropivacaine is a frequently employed local anesthetic in clinical settings, utilized for surgical anesthesia and acute pain management. The findings of the study indicate that ropivacaine effectively reduces ALDH positive cells, diminishes the sphere formation capacity of SKOV3 cells, lowers the protein levels of OCT4 and Nanog, and inhibits the stemness of ovarian cancer cells by deactivating the PI3K/AKT signaling pathway. Furthermore, ropivacaine has been demonstrated to expedite ferroptosis in ovarian cancer cells (Lu et al., 2022).

Studies have demonstrated the potential of mTOR (LY-294002 and sirolimus) and PI3K inhibitors (wortmannin) as ferroptosis-inducing drugs (Zhang J. et al., 2021). Additionally, a preclinical investigation has confirmed that the PI3K-AKT-mTOR pathway suppresses ferroptosis, and that inhibiting PI3K and mTOR triggers ferroptosis in ovarian cancer cells (Yi et al., 2020b). These findings establish a theoretical foundation for the clinical use of mTOR and PI3K inhibitors in the treatment of ovarian cancer.

Despite incomplete comprehension of the precise cellular ferroptosis mechanism, lipid peroxidation has been recognized as a crucial process in ferroptosis. Considering the significance of lipid metabolism in ferroptosis and the essential function of lipids in ovarian cancer, stearoyl-coa desaturase (SCD1, SCD) is an enzyme that facilitates the rate-limiting step in the production of monounsaturated fatty acids in ovarian cancer cells. SCD1 is expressed at high levels in ovarian cancer tissues, cell lines, and genetic models of ovarian cancer stem cells. The inhibition of stearoyl-CoA desaturase 1 (SCD1) has been shown to elicit ferroptosis and apoptosis, and its inhibition can significantly augment the anti-tumor efficacy of ferroptosis inducers. The concurrent administration of SCD1 inhibitors and ferroptosis inducers presents a novel therapeutic approach for ovarian cancer patients (Tesfay et al., 2019). Squalene synthase influences various metabolites in the mevalonate (MVA) pathway, including coenzyme Q10, a lipophilic antioxidant, and its inhibition can sensitize cells to ferroptosis (Shimada et al., 2016). Collectively, the modulation of cellular lipid composition may disrupt ferroptosis.

The expression of HIC1 is notably reduced in ovarian cancer patients, whereas it is significantly elevated in high-risk patients with unfavorable prognoses. It is postulated that the divergent impacts of HIC1 on ovarian tumorigenesis and patient outcome may be attributed to its regulation of biological processes in distinct contexts via distinct mechanisms. Research has substantiated that the downregulation of HIC1 augments the efficacy of chemotherapy and immunotherapy in individuals with ovarian cancer by instigating ferroptosis (Wang H. et al., 2022). This suggests that HIC1 may play a crucial part in the management of ovarian cancer via the mediation of ferroptosis, and thus, may serve as a promising therapeutic target for ovarian cancer.

The enzyme Methylenetetrahydrofolate reductase (MTHFR) plays a crucial role in folate metabolism (Weile et al., 2021). Recent research has revealed that MTHFR can impede the ubiquitination and degradation of HMOX1 by competitively interacting with TRC8, thereby hindering the onset of ferroptosis in ovarian cancer cells and facilitating the growth of tumor cells (Wang X. et al., 2022). Consequently, these discoveries will establish the groundwork for the creation of innovative therapeutic agents that aim at MTHFR for the purpose of diagnostic and therapeutic intervention in patients with ovarian cancer.

The regulatory effect of LncRNA CACNA1G-AS1 on ferroptosis through FTH1-IGF2BP1 has been found to promote the malignant phenotype of ovarian cancer cells (Jin et al., 2023). This discovery highlights the potential of LncRNA CACNA1G-AS1 as a therapeutic target for ovarian cancer treatment.

The natural compound MAP30, derived from non-toxic bitter gourd, has been found to exhibit anti-ovarian cancer properties by activating AMPK via the AMP-independent CaMKKβ pathway and inhibiting mTOR and the novel AKT/ERK/FOXM1 signaling pathway. Additionally, MAP30 has been identified as an inducer of ferroptosis in ovarian cancer cells. Although the influence of AMPK on MAP30-induced ferroptosis in ovarian cancer cells has been established, the precise mechanism underlying this relationship requires further investigation (Chan et al., 2020). Significantly, the activities of MAP30 augment its anticancer properties and mitigate chemotherapy resistance in ovarian cancer, thereby substantiating its potential as a supplementary agent to enhance the effectiveness of chemotherapy protocols in the treatment of ovarian cancer.

According to a recent study, frizzled7 has been found to impede ferroptosis in both *in vitro* and *in vivo* models of ovarian cancer, while also fostering stemness traits in ovarian cancer cells, thus hindering the effectiveness of ovarian cancer treatment (Wang et al., 2021a). This finding implies that frizzled7 could serve as a promising therapeutic target for ovarian cancer.

Recent evidence has emerged indicating that the induction of ferroptosis may serve as a viable strategy to overcome drug resistance in cancer treatment, particularly in tumors that exhibit resistance to conventional therapies (Zhang C. et al., 2022). A promising agent in this regard is recombinant human cyst(e)inase, which can effectively initiate cellular ferroptosis by depleting plasma cystine (Cramer et al., 2017). Its anti-tumor efficacy and *in vivo* safety have been established in a range of tumor mouse models, thereby suggesting its potential to trigger ferroptosis in ovarian cancer cells and improve clinical outcomes.

According to research findings, ADAMTS9-AS1 functions as a suppressor of ferroptosis in ovarian cancer by directing its focus towards the miR-587/SLC7A11 axis. This discovery presents a novel therapeutic target for ovarian cancer treatment and serves as a valuable reference for comprehending the molecular mechanism of ferroptosis in ovarian cancer cells (Cai et al., 2022).

According to recent reports, the over-activation of CEBPG plays a crucial role in the promotion of cancer development (Huang et al., 2020b). This transcription factor impedes the ferroptosis of tumor cells by up-regulating SLC7A11, which is a negative regulator of ferroptosis, thereby hastening the progression of ovarian cancer (Zhang X. et al., 2023). Therefore, CEBPG is a significant transcriptional regulator of ferroptosis and a prognostic factor that holds potential therapeutic value.

FZD7 has the potential to serve as a novel marker for tumor cell populations that exhibit an abundance of antioxidant response mechanisms. These cells, which possess stemness characteristics and are responsible for disease recurrence following chemotherapy, can be effectively eliminated through the activation of the ferroptosis pathway (Wang et al., 2021b). As such, the targeting of Pt-T FZD7þ cells through the induction of ferroptosis represents a promising therapeutic strategy for the treatment of ovarian cancer.

O- glycosylation, a prevalent protein modification mechanism, is initiated by GALNT, which is situated in the Golgi apparatus (Carraway et al., 2007). A growing body of evidence indicates that GALNT plays a role in tumor progression by catalyzing specific substrates (Wang et al., 2013). Specifically, GALNT14 is implicated in the EGFR/mTOR pathway, and the suppression of GALNT14 activity leads to a reduction in mTOR activity, resulting in decreased levels of SLC7A11 and GPX4, ultimately leading to the induction of ferroptosis. The concomitant administration of mTOR inhibitors and cisplatin resulted in an additive impact on ovarian cancer cells by inducing apoptosis and ferroptosis (Li et al., 2022). This observation implies that the combined use of cisplatin and mTOR inhibitors could be a viable approach to surmount cisplatin resistance in ovarian cancer treatment.

Hydroquinidine (HQ), a naturally occurring alkaloid, is utilized in the treatment of cardiac arrhythmias and Brugada syndrome. Its mechanism of action involves the alteration of the ionic gradient and membrane potential as an ion channel blocker. The potential anti-tumor properties of ion channel blockers have been increasingly supported by evidence (Altamura et al., 2022), and the inhibition of tumor growth through ion channel blockade represents a promising therapeutic strategy. The study revealed that HQ treatment inhibited the cell cycle of ovarian cancer cells and activated the apoptosis and ferroptosis pathways (Yavuz et al., 2023). Consequently, HQ exhibits potential as a therapeutic agent for ovarian cancer.

The balance of lipid metabolic activity and redox-driven ferroptosis in ovarian cancer cells derived from ascites is influenced by the SCD1/FADS2 fatty acid desaturation enzyme, as reported in a study (Xuan et al., 2022). The combination of SCD1/FADS2 inhibitors with cisplatin has been found to exhibit a synergistic effect in inhibiting the dissemination of ovarian cancer cells, thereby offering a promising chemotherapy strategy for the treatment of peritoneal metastasis of ovarian cancer.

The augmentation of the intracellular labile iron pool (LIP) represents a promising yet underexplored approach to induce ferroptosis in neoplastic cells. Research has demonstrated that the regulation of ferritin synthesis and degradation is under the control of NRF2, which governs HERC2 (an E3 ubiquitin ligase of NCOA4 and FBXL5) and VAMP8 (a mediator of autophagosome-lysosome fusion), thereby modulating intracellular iron homeostasis. The viability of inhibiting NRF2 to elevate LIP levels and induce ferroptotic death in ovarian cancer cells has been confirmed in preclinical models (Anandhan et al., 2023).

Norcantharidin (NCTD), a demethylating derivative of cantharidin, has been extensively employed as an alternative therapeutic agent for cancer in clinical settings. Research has demonstrated that NCTD elicits ferroptosis by suppressing the NRF2/HO-1/GPX4/xCT axis signaling in ovarian cancer cells (Zhu et al., 2022). Consequently, NCTD has been established as a promising therapeutic option for ovarian cancer treatment.

Numerous investigations have demonstrated that peroxisomes facilitate the occurrence of ferroptosis through the production of polyunsaturated ether phospholipids (PUFA-ePLs), which serve as substrates for lipid peroxidation and ultimately lead to ferroptosis (Zou et al., 2020b). This highlights the significance of the peroxisome-ether-phospholipid axis in influencing the susceptibility of cells to ferroptosis, underscores the distinctive functional lipid class of PUFA-ePLs that undergoes dynamic regulation during cellular state transitions, and implies the existence of multiple regulatory nodes that can be targeted for therapeutic intervention in ovarian cancer.

ALOX12 serves as the primary gene responsible for driving ferroptosis, and its mechanism of action involves the conversion of polyunsaturated fatty acids, particularly arachidonic acid, into the bioactive lipid (12S)-hydroperoxide eicosapentaenoic acid (Zheng et al., 2020). The lipogenesis induced by Alox12 plays a crucial role in the process of ferroptosis (Cheng et al., 2023). Furthermore, ALOX12 exhibits a clinical predictive value in ovarian cancer patients, indicating its potential as a prognostic tool and therapeutic target for this patient population.

Menin-MLL inhibitors exhibit potential as therapeutic agents for leukemia characterized by MLL rearrangement. Research has demonstrated that the combination of Menin-MLL inhibitors, such as MI-463, with auranofin to induce ferroptosis is an efficacious treatment strategy for various cancers, including ovarian cancer (Kato et al., 2020).

The co-administration of olaparib and arsenic trioxide (ATO) was found to induce lipid peroxidation and subsequently initiate ferroptosis. The underlying mechanism involves the activation of the AMPKα pathway and the suppression of stearoyl-coa desaturase 1 (SCD1) expression by the combination of ATO and olaparib (Tang et al., 2022). These findings suggest that ATO treatment can augment the efficacy of olaparib against platinum-resistant ovarian cancer (refer to Table 1).

**TABLE 1:** Therapies targeting ferroptosis-related signaling pathways and genes.

Drug	Targeting signaling pathways or genes	Mechanism	References
erastin	k-ras gene mutation/ABCB1	Induction of ferroptosis	Zhou et al. (2019a), Mukhopadhyay et al. (2021)
ART	ROS	Promotes strong induction of reactive oxygen species	Greenshields et al. (2017)
olaparib	SLC7A11	Promotes cell ferroptosis	Hong et al. (2021a)
SNAI2	SLC7A11	Promotes cell ferroptosis	Fan et al. (2020), Jin et al. (2022a)
SPIONs	Induction of oxidative stress	Promotes cell ferroptosis	Huang et al. (2020a)
MEX3A	p53	Prevent cell ferroptosis	Wang et al. (2023)
YAP/SKP2 stimulator	Cell proliferation program and susceptibility to ferroptosis	Promotes cell ferroptosis	Cai et al. (2020), Yang et al. (2021)
carboplatin	ACSL1/FSP1	Resistance to ferroptosis	Zhang et al. (2023a)
SPIO-Serum	p53	Promotes cell ferroptosis	Zhang et al. (2021a)
sEV	mediates intercellular communication	Promotes cell ferroptosis	Yu et al. (2019a), Brown et al. (2019)
targeted drugs	PKCII-ACSL4/PI3K-AKT-mTOR	Resistance to ferroptosis	Yi et al. (2020a), Zhang et al. (2022a)
TAZ	ANGPTL4 - NOX2	Promotes cell ferroptosis	Yang et al. (2020)
Reduce miR-4715-3p/miR-9/miR-137	Promotes cell ferroptosis	Promotes cell ferroptosis	Berindan-Neagoe et al. (2014), Bagnoli et al. (2016), He et al. (2019)
Increase miR-424-5p	restrain ACSL4	Reduced cell ferroptosis	Ma et al. (2021)
Apatinib plus olaparib	p53	Promotes cell ferroptosis	Liang et al. (2019a), Kang et al. (2019)
CMP	Nrf2/HO-1/xCT/GPX4	Promotes cell ferroptosis	Jin et al. (2022b)
FINs	SLC7A11-GSH	Promotes cell ferroptosis	Dixon et al. (2014a), Stockwell et al. (2017a)
lidocaine	SLC7A11	Promotes cell ferroptosis	Sun et al. (2021)
ropivacaine	Promotes cell ferroptosis	Promotes cell ferroptosis	Lu et al. (2022)
mTOR/PI3K inhibitor	PI3K- AKT -mTOR	Promotes cell ferroptosis	Yi et al. (2020b), Zhang et al. (2021b)
SCD1	Promotes cell ferroptosis	Promotes cell ferroptosis	Tesfay et al. (2019)
squalene synthetase	Mevalonate pathway	Increased ferroptosis sensitivity	Shimada et al. (2016)
Target HIC1	HIC1	Promotes cell ferroptosis	Wang et al. (2022a)
Target MTHFR	MTHFR	Promotes cell ferroptosis	Wang et al. (2022b)
LncRNA CACNA1G-AS1	FTH1-IGF2BP1	regulates cell ferroptosis	Jin et al. (2023)
MAP30	AKT/ERK/FOXM1	Promotes cell ferroptosis	Chan et al. (2020)
frizzled7	Inhibition of cell ferroptosis	Inhibits cell ferroptosis	Wang et al. (2021a)
recombinant human cystinase(e)	Consumption of plasma cystine	Trigger cell ferroptosis	Cramer et al. (2017)
ADAMTS9-AS1	miR-587/SLC7A11	Inhibition of cell ferroptosis	Cai et al. (2022)
CEBPG	SLC7A11	Inhibition of cell ferroptosis	Zhang et al. (2023b)
Target FZD7	FZD7	Promotes cell ferroptosis	Wang et al. (2021b)
Cisplatin was combined with an mTOR inhibitor	SLC7A11/GPX4	Promotes cell ferroptosis	Li et al. (2022)
HQ	Activation of ferroptosis	Promotes cell ferroptosis	Yavuz et al. (2023)
SCD1/FADS2 inhibitor	Promotes cell ferroptosis	Promotes cell ferroptosis	Xuan et al. (2022)
Target NRF2	HERC2/VAMP8	Control of iron homeostasis	Anandhan et al. (2023)
NCTD	NRF2/HO - 1/GPX4/xCT	Induction of ferroptosis	Zhu et al. (2022)
PUFA-ePLs	The peroxisome -ether-phospholipid axis	Promotes cell ferroptosis	Zou et al. (2020b)
Target ALOX12	Driven iron death	Promotes cell ferroptosis	Cheng et al. (2023)
menin-MLL inhibitor	Driven iron death	Promotes cell ferroptosis	Kato et al. (2020)
Olaparib in combination with arsenic trioxide	Activated AMPKα pathway	Promotes cell ferroptosis	Tang et al. (2022)

### 3.2 Ferroautophagy and ferroptosis

Recent research has demonstrated that ferroptosis is reliant upon autophagy, as the latter process can induce ferroptosis by degrading ferritin, leading to an increase in iron levels and lipid ROS(Hou et al., 2016). Chaperone-mediated autophagy (CMA) is a form of selective autophagy that involves lysosomal degradation of intracellular components. BECN1 is a protein that plays a crucial role in promoting autophagy. Recent investigations have revealed that BECN1 can directly impede systemic XC-activity by binding to SLC7A11, thereby facilitating ferroptosis in ovarian cancer cells (Song et al., 2018). The findings indicate that autophagy serves to facilitate ferroptosis. Nevertheless, contemporary research has revealed that the administration of metformin can trigger ferroptosis by counteracting the generation of lipid ROS through autophagy inhibition (Chen et al., 2022). The complex interplay between ferroptosis and autophagy remains a topic of ongoing investigation.

Nuclear receptor coactivator 4 (NCOA4) is a discerning receptor that upholds iron homeostasis by binding to ferritin, conveying ferritin to lysosomes, and stimulating autophagic degradation. Recent investigations have revealed that NCOA4 is a constituent of autophagosomes that participates in the autophagic process of ferritin (Zhang et al., 2021c). In 2014, Mancias substantiated that NCOA4 is a distinct receptor for ferritin autophagy via proteomic analyses, thereby establishing a connection between cellular autophagy and ferroptosis (Mancias et al., 2014). Subsequent research has demonstrated that ferritin autophagy plays a crucial role in regulating cell ferroptosis. Specifically, Masaldan et al. have established a close relationship between iron accumulation, impaired ferritin autophagy in aging cells, and the inhibition of ferroptosis (Masaldan et al., 2018). Additionally, Gao et al. have reported that the inhibition of NCOA4 expression leads to the inhibition of ferritin autophagy, resulting in the accumulation of Fe2+ and the formation of lipid peroxides, which are associated with ferroptosis (Gao et al., 2016). Subsequent experiments have demonstrated that C-MYC has the ability to impede ferroptosis through direct targeting of NCOA4, resulting in the downregulation of NCOA4 expression. Additionally, C-MYC can foster the malignant phenotype and immune evasion of ovarian cancer cells by suppressing ferritin autophagy (Jin et al., 2022c). These findings offer a novel therapeutic approach for the treatment of ovarian cancer.

Research has demonstrated that the augmentation of NCOA4-mediated autophagic degradation of ferritin, commonly referred to as ferroautophagy, induces the accumulation of labile iron pool (LIP) and reactive oxygen species (ROS), ultimately leading to the occurrence of cell ferroptosis (Del Rey and Mancias, 2019). Conversely, the activation of the NRF2 pathway or the inhibition of the serine/threonine kinase ATM impedes cellular ferroptosis by enhancing cellular iron stores through the transcriptional activation of ferritin heavy chain (FtH) (Chen et al., 2020). These findings suggest that the regulation of ferroptosis via ferroautophagy may hold potential therapeutic implications for ovarian cancer.

Research has substantiated that the expression of CACNA1G-AS1 is considerably elevated in ovarian cancer tissues and induces upregulation of FTH1 via IGF2BP1-mediated m6A methylation. CACNA1G-AS1 impedes ferroptosis in ovarian cancer cells and fosters the malignant phenotype of cells through IGF2BP1-FTH1-mediated ferroautophagy, thereby presenting a novel biomarker and therapeutic target for the clinical assessment of ovarian cancer patients (Jin et al., 2023).

### 3.3 Immunotherapy and ferroptosis

Despite the persistent endeavors of scholars to investigate treatment strategies for ovarian cancer, the available options for treatment remain limited, with high rates of recurrence and chemotherapy resistance. Immunotherapy is a promising avenue for cancer treatment, with previous research indicating a close association between ferroptosis and tumor immunotherapy (Wang et al., 2019b; Lang et al., 2019). Furthermore, evidence suggests that ovarian cancer is an immunogenic tumor (Kandalaft et al., 2011). Immune checkpoint blockade, which is considered one of the most influential immunotherapies, has garnered growing interest from researchers who are targeting aspects related to ferroptosis (Stockwell and Jiang, 2019). Research has demonstrated that the combination of ferroptosis inducers and immune checkpoint blockade synergistically enhances T cell-mediated antitumor immunity and ferroptosis (Wang et al., 2019b).

It has been posited that, akin to apoptosis, ferroptosis may elicit the recruitment of immune cells to its site via the presentation of antigens facilitated by the release of “Find Me” signals. *In vitro* conditions have demonstrated the phagocytosis of these cells by macrophages. Contemporary research indicates that neoplastic cells undergoing ferroptosis may also discharge DAMPs into the extracellular milieu through autophagy, thereby enlisting and stimulating immune cells. These mechanisms have been observed across diverse models. For instance, in a myocardial ischemia-reperfusion injury model, ferroptotic cells can trigger immunity and attract neutrophils by discharging DAMPs. In a pancreatic cancer model, ferroptotic tumor cells secrete KRAS, which leads to macrophage immunosuppression and fatty acid oxidation. Additionally, various investigations have indicated that ferroptotic tumor cells can release substantial quantities of prostaglandin E2 (PGE2), which could be associated with the elevation of prostaglandin endoperoxide synthase 2 (PTGS2) expression. Research has established that PGE2 exhibits immunosuppressive properties and functions as an inflammatory factor. In light of these discoveries, scholars have postulated that tumor cells undergoing ferroptosis may trigger the adaptive immune system by discharging particular factors (HMGB1 and PGE2), attract immune cells to their vicinity, and consequently effectuate the eradication of malignant cells (Angeli et al., 2019; Xu et al., 2021; Shi et al., 2022).

**FIGURE 2:**
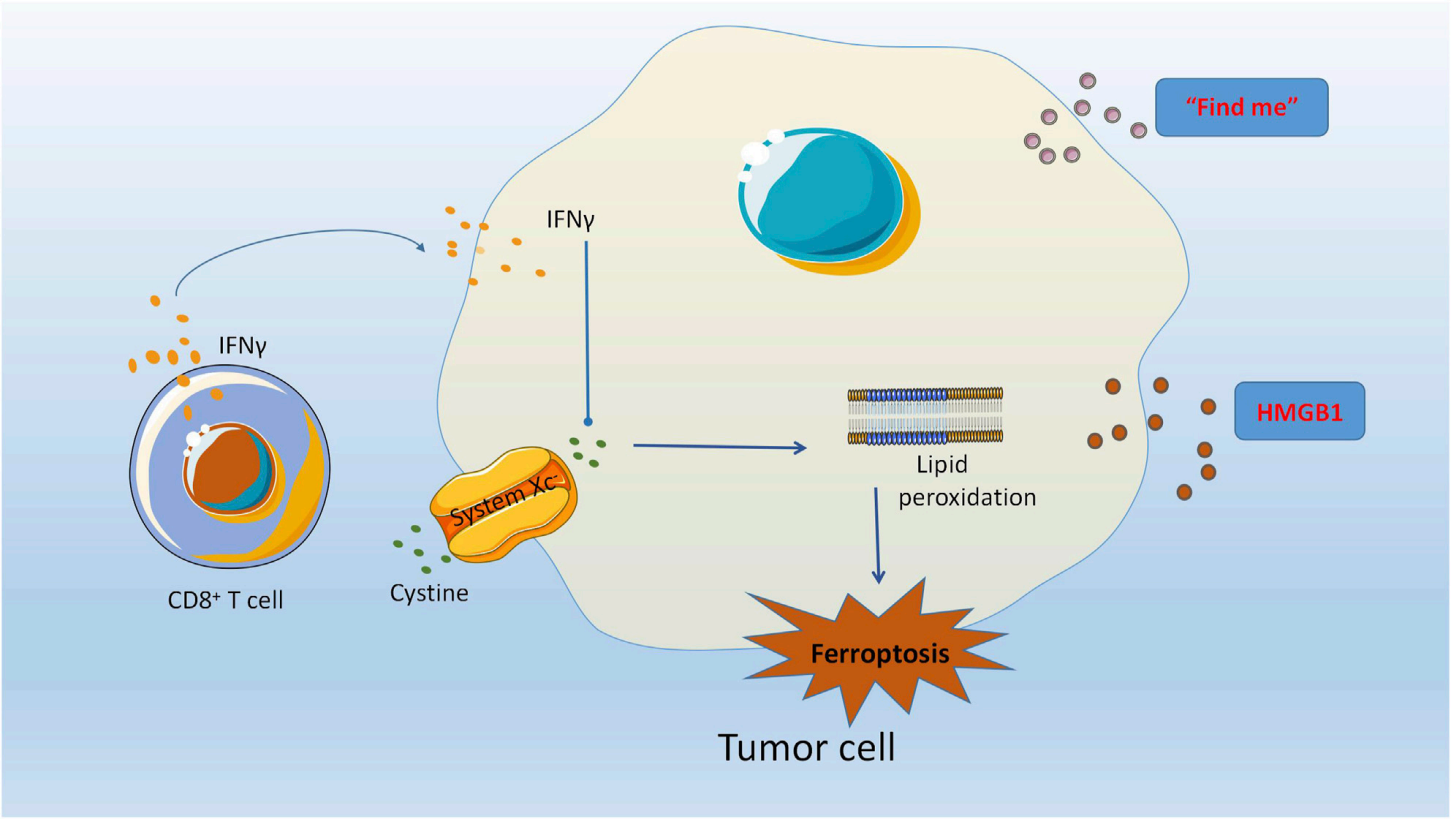
The modulation of ferroptosis by immunotherapy has been shown to augment the effector capabilities of CD8 (+) T cells, thereby increasing the susceptibility of tumor cells to therapeutic interventions. The autophagy-mediated release of high mobility group box 1 protein (HMGB1) from ferroptotic cells plays a crucial role in the induction of immunogenic cell death (ICD) in cancer cells, as HMGB1 serves as a critical damage-associated molecular pattern (DAMP). CD8^+^ T cells release Interferon-γ (IFNγ) which can effectively downregulate the expression of SLC3A2 and SLC7A11, two subunits of the glutamate-cystine antiporter system Xc -. This, in turn, weakens the cystine uptake by tumor cells, promoting lipid peroxidation and ferroptosis of tumor cells.

Wang et al. discovered that the concurrent implementation of ferroptosis induction and immunotherapy can result in a synergistic enhancement of the anticancer effect (Wang et al., 2019a). The mechanism underlying this phenomenon has been extensively investigated, revealing that immunotherapy-triggered CD8^+^ T cells can induce ferroptosis in tumor cells. Consequently, immunotherapy can modulate the ferroptosis process to augment the effector function of CD8^+^ T cells and heighten the sensitivity of tumor cells to treatment. Furthermore, the release of high mobility group protein B1 (HMGB1) by ferroptotic cells occurs in an autophagy-dependent manner (Wen et al., 2019). HMGB1, a crucial protein for immunogenic cell death (ICD) of cancer cells, serves as a significant damage-associated molecular pattern (DAMP) (Garg et al., 2015). Ferroptosis has the potential to elicit a robust immune response via ICD, thereby augmenting anti-tumor immunity. Additional research has demonstrated that immunotherapy-activated CD8^+^ T cells enhance ferroptosis-specific lipid peroxidation in tumor cells, and the induction of cell ferroptosis contributes to the anti-tumor effectiveness of immunotherapy. The mechanism by which interferon-γ (IFNγ) released by CD8^+^ T cells exerts its effects involves the downregulation of SLC3A2 and SLC7A11, two subunits of the glutamic acid-cystine antitransporter system Xc -. This downregulation weakens the uptake of cystine by tumor cells, promoting lipid peroxidation and ferroptosis of tumor cells (Wang et al., 2019a). Additionally, TYRO3 has been found to be highly expressed in anti-PD-1/PD-L1 treatment of tumors such as ovarian cancer, and can inhibit tumor cell ferroptosis, as noted in another article. The findings indicate that ferroptosis plays a crucial role in the immunotherapeutic approach to combat ovarian cancer. Enhanced comprehension of the underlying biological mechanisms and identification of pivotal targets are anticipated to offer novel therapeutic avenues for ovarian cancer. A promising treatment modality is the integration of targeted signaling pathways with immune checkpoint blockade (refer to Figure 2).

## 4 Prognostic prediction of ferroptosis-related genes

Although diagnostic techniques for ovarian cancer have advanced in recent decades, the diagnosis and prognostic prediction of patients with this disease remain inadequate. Currently, recognized risk factors for ovarian cancer comprise genetic predisposition, obesity, advanced age, and perineal talc use (Penninkilampi and Eslick, 2018; Lheureux et al., 2019b). However, these factors have not proven to be reliable predictors of patient prognosis. In the past few years, the identification of ferroptosis-associated genes has led to their potential use as prognostic indicators for various tumors, such as breast cancer, hepatocellular carcinoma, lung cancer, melanoma, and ovarian cancer (Du and Zhang, 2020; Piatek et al., 2020; Zhang J. et al., 2021; Wang et al., 2021c; Xu and Chen, 2021; Yao et al., 2021; Zhu et al., 2021). Several studies have reported the development of prognostic prediction signatures based on ferroptosis-related genes (FRGs) in ovarian cancer, highlighting the pressing need for novel biomarker models to aid in the diagnosis and prognosis of this disease.

Previous research has indicated that the expression of LOC390705 was downregulated, while KLHL24 and GPT2 expression was upregulated in HT-1080 cells treated with erastin. Additionally, SETD1B was found to be enriched in GPX4 inhibitor ml162 resistant cells (Dixon S. J. et al., 2014; Dixon et al., 2015). Furthermore, HIC1(Zhang X. et al., 2019), MUC1 (Hasegawa et al., 2016), PML (Saint-Germain et al., 2017), MT1G (Sun et al., 2016), and AKR1C1 (Dixon S. J. et al., 2014; Gagliardi et al., 2019) have been identified as inhibitors of ferroptosis, while ACSF2 has been identified as a driver of ferroptosis (Dixon S. J. et al., 2012). The utilization of these 10 genes in a diagnostic model for ovarian cancer has demonstrated high sensitivity and specificity, thereby aiding in the early detection of ovarian cancer. Researchers have identified three genes (HIC1, LPCAT3, DUOX1) associated with ferroptosis that can be used to predict the prognosis of ovarian cancer patients (Wang H. et al., 2022). Through the use of various bioinformatics methods, genes and eight significant lncRNAs linked to ferroptosis in ovarian cancer cells have been screened and identified. These lncRNAs include RP11-443B7.3, RP5-1028K7.2, TRAM2-AS1, AC073283.4, RP11-486G15.2, RP11-95H3.1, RP11-958f21.1, and AC006129.1 (Wang et al., 2021d; Wang Z. et al., 2021). The aforementioned ferroptosis-related genes and significant lncRNAs may serve as crucial clinical biomarkers. The identification and targeting of specific biomarkers presents a promising avenue for the diagnosis, clinical management, and prognostic assessment of ovarian cancer. Notably, patients exhibiting elevated levels of ferroptosis-related long non-coding RNA may derive greater benefit from conventional chemotherapy or ferroptosis-inducing therapy. Furthermore, certain scholars have conducted screenings of immune genes (CXCL11, CX3CR1) and ferroptosis genes (FH, DNAJB6), culminating in the development of a prognostic prediction model (Yuan et al., 2022). The four genes identified in this study hold promise as biomarkers for ferroptosis and may be utilized in immune drug therapy, personalized treatment, efficacy prediction, and follow-up planning. Notably, a prognostic grading model for ovarian cancer has been established by certain scholars, incorporating five ferroptosis-associated factors (ALOX12, ACACA, SLC7A11, FTH1, CD44) through biological analysis. This model has demonstrated significant value in the analysis of disease prognosis. A distinct investigation established a 15-gene prognostic signature through the screening of genes implicated in ferroptosis within ovarian cancer cells. The 15 genes, which include ferroptosis driver genes (CYBB, VDAC2, SOCS1, LINC00472, ELAVL1, IFNG, IDH1), ferroptosis suppressor genes (NRAS, MT1G, ACSL3, SLC3A2), and ferroptosis markers (PTGS2, SLC1A4, PCK2, XBP1), can be broadly categorized into four groups Class 3 (Liu et al., 2022). These genes play a crucial role in the development and progression of various tumors, including ovarian cancer. Furthermore, a prognostic signature consisting of 11 ferroptosis-related long non-coding RNAs (lncRNAs) was able to accurately predict the prognosis of patients with ovarian cancer. This signature, which includes AC007848.1, AC011445.1, AC093895.1, AC010336.5, AL157871.2, AP001033.1, AC009403.1, AC068792.1, LINC01857, LINC00239, and AL513550.1, has been identified as an independent prognostic factor in ovarian cancer and is significantly correlated with clinical features (Peng et al., 2021). A recent study has proposed a set of eight FIRLs tags, namely AC138904.1, AP005205.2, AC007114.1, LINC00665, UBXN10-AS1 AC083880.1, LINC01558, and AL023583.1, which may serve as a useful tool for guiding the prognosis and treatment decisions of ovarian cancer patients (Feng et al., 2022). Additionally, some scholars have identified and validated a novel ferroptosis-related gene signature, consisting of SLC7A11, ZFP36, and TTBK2, as an independent prognostic indicator for predicting the treatment response of ovarian cancer patients (Yang et al., 2023). This discovery offers novel perspectives on the ferroptosis patterns and immune infiltration in ovarian cancer, thereby facilitating tailored treatment decisions through a promising prognostic signature for treatment response and prognosis. Prior research has demonstrated that STEAP3, functioning as a metal reductase, transforms Fe3+ into Fe2+ and contributes to iron homeostasis in tissues and cells. STEAP3 has been identified as being upregulated in ovarian cancer cells, and its potential as a prognostic marker for ovarian cancer patients has been validated (Cheng et al., 2023).

In recent times, there has been a surge in research regarding the utilization of ferroptosis in cancer, with a particular focus on its prognostic function in tumors such as ovarian cancer, which is still in its nascent stages. The identification of biomarkers is crucial in elucidating the precise mechanisms of ferroptosis and immune response in ovarian cancer cells, and holds immense significance in predicting the prognosis of patients afflicted with ovarian cancer. Nevertheless, it is noteworthy that the prognostic models constructed in these studies were based on a restricted number of FRGs and lacked direct validation in clinical tissues of ovarian cancer patients. Numerous investigations have demonstrated the interplay between immunity and ferroptosis in ovarian cancer cells, which presents novel avenues for the clinical management of ovarian cancer patients’ treatment and prognosis.

## 5 Summary and prospect

Ovarian cancer is a tumor characterized by high heterogeneity and a significant number of somatic mutations, resulting in the highest mortality rate among all gynecologic malignancies (Yang et al., 2017). This disease poses a significant challenge to women worldwide, with a high morbidity rate. In recent years, there has been a concerted effort to uncover the etiology and pathogenesis of ovarian cancer, with the aim of expanding treatment strategies. The selective induction of ovarian cancer cell death represents a promising approach for treating this malignant tumor (Liu H. J. et al., 2020). According to current knowledge, there is mounting evidence indicating that ferroptosis is a pivotal factor in the advancement and management of ovarian cancer. Nevertheless, the quantity of research pertaining to ferroptosis in ovarian cancer cells is limited and necessitates further exploration. The aberrant regulation of the cellular death mechanism is a critical determinant in the onset and advancement of neoplastic growth. It is well-established that the resistance to apoptosis is a hallmark feature of cancer development and progression (Frank et al., 2019; Koren and Fuchs, 2021). Over the past few decades, scholars have dedicated their efforts towards identifying and comprehending the regulatory mechanisms of non-apoptotic forms of cell death, with the aim of enhancing cancer diagnosis and treatment. Ferroptosis, a recently discovered regulatory cell death (RCD), has emerged as a significant player in various diseases, including ovarian cancer, and has garnered increasing attention from scholars. As a finely regulated cell death process, ferroptosis may serve as an adaptive mechanism to eliminate malignant cells. The potential clinical utility of ferroptosis in cancer therapy has been explored.

Research has indicated that specific cancer cells demonstrate a notable dependence on iron, rendering iron-dependent cancer cells more vulnerable to ferroptosis (Brown et al., 2020). Consequently, targeting ferroptosis may present a promising avenue for treating ovarian cancer. Despite the potential for inducing cell ferroptosis to yield significant anti-tumor effects, obstacles persist. The precise tumor types that respond to ferroptosis inducers remain unclear, although TAZ-activated tumors may exhibit heightened sensitivity to various ferroptosis-inducing therapies. Hence, the activation of TAZ and the consequent induction of its canonical target genes can serve as a reliable predictive biomarker (Yang et al., 2020). Furthermore, it is imperative to evaluate the efficacy of compounds or therapeutic agents that can effectively induce ferroptosis in cells. While erastin and various GPX4 inhibitors have demonstrated the ability to induce ferroptosis, their toxicity and stability may impede their *in vivo* application and clinical translational potential. To date, numerous ferroptosis-inducing agents have been identified and sanctioned, with the aim of surmounting the limitations of conventional chemotherapeutic agents that trigger apoptosis (Hassannia et al., 2019b; Cosialls et al., 2021). However, the rational clinical implementation of FINs is still beset by certain challenges that are arduous to overcome. These agents exhibit physical and chemical deficiencies, including inadequate pharmacokinetics, low aqueous solubility, and, most notably, metabolic instability (Lin et al., 2020; Nie et al., 2022). The FDA-approved class I FIN sulfasalazine (SAS) has been shown to induce cell ferroptosis in breast and head and neck cancers by inhibiting SLC7A11, as evidenced by studies (Kim et al., 2018; Yu H. et al., 2019). However, its clinical efficacy in treating ovarian cancer is limited (Tang et al., 2021). Consequently, the identification of novel drug combinations that can act synergistically with FINs on ovarian cancer cells represents a promising avenue for addressing this issue.

Platinum-based chemotherapy has been observed to trigger apoptosis and ferroptosis in ovarian cancer cells through the release of a significant quantity of ROS, which subsequently causes damage to the DNA or cell membrane (Perillo et al., 2020). The induction of cell ferroptosis has been found to have either antitumor effects or to enhance the chemosensitivity of ovarian cancer cells (Li et al., 2021). However, extensive research in this area has revealed that different ovarian cancer cells exhibit varying levels of susceptibility to cellular ferroptosis. Therefore, when employing ferroptosis inducers as novel therapeutic approaches for ovarian cancer, these factors must be taken into consideration.

Research has substantiated that chemotherapy can induce hormonal imbalances and harm the ovaries. Additionally, studies have demonstrated that chemotherapy medications trigger ferroptosis in ovarian cells by means of an overabundance of ROS-induced lipid peroxidation and mitochondrial dysfunction, ultimately resulting in ovarian damage (Zhang S. et al., 2023). Consequently, the creation of fertility-preserving agents that target chemotherapy-induced oxidative stress and ferroptosis has the potential to mitigate ovarian damage and enhance the overall wellbeing of individuals with cancer. The extensive utilization of radiotherapy in the realm of cancer therapy has garnered increasing attention towards radiation-induced reproductive system impairment. The irreversible harm inflicted by radiation on female ovarian function underscores the significance of enhancing comprehension of the mechanism of radiation-induced ovarian injury and investigating potential pharmacological protective measures. Radiation has been observed to diminish the viability of ovarian granulosa tumor cells (KGN), alter the morphology of mitochondria, trigger intracellular iron accumulation, heighten oxidative stress, and induce lipid peroxidation. Furthermore, radiation has been found to elicit ferroptosis in KGN cells. Sphingosine-1-phosphate (S1P), a crucial bioactive sphingolipid, has been shown to mitigate radiation-induced ferroptosis and confer protection against ovarian injury. However, the protective effect of S1P was found to be nullified upon siRNA-mediated knockdown of glutathione peroxidase 4 expression. The prevalence of ferroptosis in radiation-induced ovarian injury suggests that S1P may serve as a protective agent against radiation damage by inhibiting cell ferroptosis (Zhao et al., 2023). Previous research has demonstrated the protective effects of S1P and its analogue fingolimod on ovarian function during chemotherapy and radiotherapy (Li et al., 2014). However, the potential benefits and risks of utilizing ferroptosis modulators to safeguard female fertility necessitate careful evaluation.

Despite significant advancements in treatment strategies for ovarian cancer in recent years, the prognosis for patients remains poor due to the active proliferation of cancer cells. Consequently, the identification of new biomarkers is crucial for evaluating patient survival, predicting survival time, and guiding clinical decision-making and follow-up. Scholars are dedicated to extending patient survival and providing clinical benefits through their research efforts. Scholars, particularly in the field of cancer, have extensively investigated and examined the relationship between immunotherapy and ferroptosis. The infiltration of a significant number of immune cells is an inevitable occurrence during the development and progression of tumors. Immune cells are crucial in tumorigenesis and the tumor microenvironment, and ferroptosis serves as a form of cell death for both tumor and immune cells. A growing body of research has focused on exploring the interplay between ferroptosis and immune molecules within tumors, yielding some notable advancements. This review presents an overview of genetic markers that integrate both immunity and ferroptosis. Recent investigations have demonstrated that cancer arises through diverse molecular pathways and mechanisms, with inherent interactions between various molecules and signaling pathways. The utilization of multi-gene signatures for predicting tumor prognosis has emerged as a prominent area of inquiry among scholars due to its reliability and precision.

Despite the promising therapeutic potential of ferroptosis induction for cancer, there is still a significant gap in knowledge regarding the genetic determinants and underlying mechanisms of this process. A comprehensive understanding of these mechanisms is crucial for identifying the tumors that are most responsive to ferroptosis-inducing agents. Numerous studies have identified several genetic determinants of ferroptosis, including oncogenic somatic mutations, regulation of iron levels, and epithelial-mesenchymal transition processes (Viswanathan et al., 2017b; Chen et al., 2019b). Within the tumor microenvironment, there exist non-genetic factors that have the potential to impact tumor progression, metastasis, and response to therapeutic interventions. These factors include, but are not limited to, tissue hypoxia, lactic acidosis, nutrient deprivation, osmotic pressure, tissue tension, and stiffness. Unlike cell-intrinsic genetic factors, the extent to which these non-genetic factors influence cellular ferroptosis remains unclear. Recent observations suggest that susceptibility to ferroptosis is significantly impacted by a non-genetic factor, namely cell density. Specifically, kidney and ovarian cancer cells have been found to exhibit heightened sensitivity to ferroptosis when cultured at low density, while displaying marked resistance to the process when grown under confluent conditions. The Hippo pathway has been identified as a participant in density-dependent ferroptosis, as it is capable of sensing and regulating the cell density-dependent phenotype through the evolutionarily conserved effectors YAP (Yes-associated protein 1) and TAZ (transcriptional coactivator PDZ-binding motif) (Yang and Chi, 2019). The inclusion of ferroptosis-inducing techniques in existing cancer treatments may enhance clinical response rates and outcomes, particularly for ovarian cancer patients with YAP/TAZ activation.

The phenomenon of ferroptosis has been observed to augment the effectiveness of immunotherapy (Wang et al., 2019c), chemotherapy (Zhou et al., 2019b), and ionizing radiation (Lei et al., 2020; Ye et al., 2020). It is anticipated that the induction of ferroptosis will emerge as a promising therapeutic approach for the management of ovarian cancer. This investigation offers significant insights into the identification of therapeutic targets and prognostic indicators associated with ferroptosis in ovarian cancer cells. Simultaneously, the examination of novel compounds as inducers and inhibitors of ferroptosis offers a valuable point of reference for the enhancement of ovarian cancer therapy. Nevertheless, the present investigation’s findings do not allow for definitive inferences regarding the clinical utility of ferroptosis in the diagnosis, treatment, and prognosis of ovarian cancer. Further investigation is required to explore inducers and inhibitors associated with ferroptosis in ovarian cancer cells, as well as the combination of novel and classical drugs, multi-drug resistance of tumor cells, and specific regulatory mechanisms. Notably, the identification of reliable predictive biomarkers for ferroptosis sensitivity is crucial to select patients with tumors who are most likely to respond. There is still much work to be done in this area. Furthermore, it is imperative to ascertain the optimal approach for inducing ferroptosis in cells and to enhance the combination strategy. Over time, we anticipate that comparable targeting of ovarian cancer patients, in conjunction with current therapies, could present a novel therapeutic pathway for ovarian cancer patients, thereby significantly enhancing the prognosis and survival rates of those with advanced ovarian cancer.
